# Molecular Response and Metabolic Reprogramming of the Spleen Coping with Cold Stress in the Chinese Soft-Shelled Turtle (*Pelodiscus sinensis*)

**DOI:** 10.3390/antiox14020217

**Published:** 2025-02-14

**Authors:** Liqin Ji, Qing Shi, Yisen Shangguan, Chen Chen, Junxian Zhu, Zhen Dong, Xiaoyou Hong, Xiaoli Liu, Chengqing Wei, Xinping Zhu, Wei Li

**Affiliations:** 1 Key Laboratory of Tropical and Subtropical Fishery Resources Application and Cultivation, Ministry of Agriculture and Rural Affairs, Pearl River Fisheries Research Institute, Chinese Academy of Fishery Sciences, Guangzhou 510275, China; jiliqin@prfri.ac.cn (L.J.); liuxl@prfri.ac.cn (X.L.);; 2 South China Sea Marine Survey Center, Key Laboratory of Marine Environmental Survey Technology and Application, Ministry of Natural Resources, Guangzhou 510275, China

**Keywords:** Chinese soft-shelled turtle, cold stress, antioxidant enzymes, transcriptome, metabolism, signaling pathways

## Abstract

The Chinese soft-shelled turtle (*Pelodiscus sinensis*), as a type of warm-water reptile, could be induced to massive death by sharp temperature decline. Hence, the mechanism of spleen tissue responding to cold stress in the *P. sinensis* was investigated. The present results showed that the superoxide dismutase (SOD) activity declined from 4 to 16 days post-cold-stress (dps), while the catalase (CAT) and glutathione peroxidase (GSH-Px) activities increased, from 4 to 8 dps in the 14 °C (T14) and 7 °C (T7) stress groups. The spleen transcriptome in the T7 group and the control group (CG) at 4 dps obtained 2625 differentially expressed genes (DEGs), including 1462 upregulated and 1663 downregulated genes. The DEGs were enriched mainly in the pathways “intestinal immune network for IgA production” (*Pigr*, *Il15ra*, *Tnfrsf17*, *Aicda*, and *Cd28*), “toll-like receptor signaling pathway” (*Mapk10*, *Tlr2*, *Tlr5*, *Tlr7*, and *Tlr8*), and “cytokine–cytokine receptor interaction” (*Cx3cl1*, *Cx3cr1*, *Cxcl14*, *Cxcr3*, and *Cxcr4*). The metabolomic data showed that esculentic acid, tyrosol, diosgenin, heptadecanoic acid, and 7-ketodeoxycholic acid were obviously increased, while baccatin III, taurohyocholate, parthenolide, enterolactone, and tricin were decreased, in the CG vs. T7 comparison. Integrated analysis of the two omics revealed that “glycine, serine and threonine metabolism”, “FoxO signaling pathway”, and “neuroactive ligand–receptor interaction” were the main pathways responding to the cold stress. Overall, this work found that low temperature remarkably influenced the antioxidant enzyme activities, gene expression pattern, and metabolite profile in the spleen, indicating that immunity might be weakened by cold stress in *P. sinensis*.

## 1. Introduction

In recent years, higher frequencies of extreme temperature events, such as cold stress, have occurred because of global climate changes and seasonal temperature variations [1]. For poikilothermic animals, such as fishes and turtles, body temperature fluctuations approximate water temperature variations. Therefore, rapid or extreme changes in water temperature may elicit a series of negative physiological alterations in aquatic animals, such as metabolic disturbance, immune suppression, and poor growth. Moreover, in certain instances, it may lead to mortality [2,3]. For example, low temperature can affect the intestinal health of the juvenile golden pompano (*Trachinotus ovatus*) [4] and the liver metabolism of orange-spotted grouper (*Epinephelus coioides*) [5]. Heat stress has influenced the immune-related gene expression and coagulation cascade pathways of the yellow pond turtle [6], as well as the antioxidative enzymes of the Chinese soft-shelled turtles [3]. Moreover, cold stress obviously influences the global fishing economy by affecting harvest yield, fishing expenditure, feed costs, and survival [7]. Therefore, studying the responsive mechanism of aquatic species to acute temperature variation is meaningful, as it protects aquatic animals’ health and reduces the aquatic industry’s financial losses.

Cold stress inhibits the immunity of aquatic animals, which might further lead to disease outbreaks in the aquaculture industry. For example, low temperature impairs the immune function of European seabass [8] and Chinese suckers [9]. Moreover, yellow pond turtles under cold stress show high mortality owing to depressed immune and metabolic functions [6]. Therefore, it is believed that the immune system of ectotherms is negatively modulated at low temperatures; hence, maintenance of immunocompetence under cold environments is challenging for some aquatic poikilotherms [2]. The immune organs carry out the main immune functions of animals. As the primary lymphatic immune organ in vertebrates from fish to mammals, the spleen plays an important part in responding to environmental stimuli and pathogenic infection [10]. For instance, heat stress induces oxidative damage in the spleen tissues of rainbow trout [11] and influences purine levels and purinergic signaling pathways in the spleen of *Brycon amazonicus* [12]. Moreover, low temperature could induce extensive tissue damage and necrosis to the spleen in Nile tilapia [13] and influence the splenic metabolism of flounder during *Edwardsiella tarda* infection [14]. Although the innate and adaptive immune systems are highly responsive to stressors in both fish and mammals, research on the role of temperature in aquatic health has often been shallow, and in-depth elucidation of key immune mechanisms remains absent [15]. In general, exploring the impact of cold stress on spleen tissue can promote elucidating the in-depth immune mechanisms of aquatic animals exposed to extreme temperatures.

Transcriptomic and metabolomic technologies have been widely used to reveal the mRNA and metabolic pattern of the spleen. Transcriptomic analysis of the spleen was performed in *Cyprinus carpio* after *Aeromonas hydrophila* infection [16], in Nile tilapia under low-temperature stress [17], and in Japanese flounder after heat stress [18]. The splenic metabolites involved in immunity have been studied in flounder after temperature alteration [14] and in largemouth bass subject to bacterial infection [19]. Moreover, combined analysis of the transcriptome and metabolome can find the most relevant pathways of specific biological processes, which may not be detectable with a single omics approach alone. For example, the integration of the two omics has been applied to explore the pathways of largemouth bass responding to *Nocardia seriolae* infection [19], kuruma shrimp under cold stress [20], and American shad exposed to high temperature [21]. However, the combination of the two omics has rarely been utilized to understand the mechanism of turtles responding to external stress.

The Chinese soft-shelled turtle is a popular economic species in Eastern Asia countries such as China, Japan, and others. The consumption of this species in China is believed to have positive effects on improving immunity, antiaging, and relieving some cardiocerebrovascular diseases [22]. Therefore, the farming industry of the Chinese soft-shelled turtle is steadily increasing in China, where approximately 497,536 tons of turtles were produced in 2023 [23]. From the end of October or November to March or April of the following year, the turtles outdoors usually experience sharp temperature variation, with possible temperature decreases of 10–20 °C in one day [24]. The acute temperature fluctuation has been reported to result in the large-scale mortality of Chinese soft-shelled turtles, bringing about substantial economic losses in the farming industry [25]. However, the molecular mechanism of immune tissues responsive to cold stress remains obscure. Hence, this study integrated biochemical, transcriptomic, and metabolomic analysis to systemically assess the molecular and metabolic patterns of the spleen in Chinese soft-shelled turtles after acute cold stress. This research can promote understanding of the responding mechanisms of spleen under low temperatures in Chinese soft-shelled turtles, providing a potential strategy for preventing cold stress-induced damage.

## 2. Materials and Methods

### 2.1. Experimental Animals

Animal treatment and experimental procedures were accomplished following the guidelines for the care and use of laboratory animals in China. The handling of the animals was approved by the Ethics Committee of the Pearl River Fisheries Research Institute, Chinese Academy of Fishery Sciences (LAEC-PRFRI-2023-10-15).

The healthy and vigorous Chinese soft-shelled turtles in the experiment were obtained from Huizhou Wealth Xing Industrial Co., Ltd. (Huizhou, China). The turtles were acclimated in a square acrylic tank (1 m × 1 m × 0.25 m) for 2 weeks before the experiments. During the acclimation, the turtles were fed commercial pellet diets provided by the Guangdong Nutriera Group Co., Ltd. (Guangzhou, China). The turtles were fed twice a day at 9:00 and 16:00 until apparent satiation. The water parameters were maintained at: temperature, 28 ± 1 °C; pH, 8.2 ± 0.4; NH_3_-N, 4.1 ± 1.1 mg/L; NO_2_^−^, 1.0 ± 0.3 mg/L; and dissolved oxygen, 5.8 ± 1.5 mg/L.

### 2.2. Cold Stress Experiment and Sample Collection

After the acclimation, a total of 540 turtles (body weight 12 ± 3 g) were randomly allocated into 18 acrylic tanks (37 cm × 25 cm × 11 cm), with each tank containing 30 individuals. These turtles were starved for 24 h before the cold stress experiment. Three groups, a 28.0 °C group (CG), a 14.0 °C group (T14), and a 7.0 °C group (T7), were set in the cold stress experiment. Each group initially had 180 turtles (30 individuals per box). RXZ-436 cooling incubators (Ningbo Jiangnan Instrument Factory, Ningbo, China) were set to decrease the temperature from 30 °C to 4 °C. After starvation, for the T14 and T7 groups, the water temperature was reduced from 28 °C to 14 °C and 7 °C at a rate of 1 °C per hour. After the cooling process, the water temperature in the CG, T14, and T7 groups were kept stable at 28 °C, 14 °C, and 7 °C, respectively, until the end of the cold stress experiment. The moment when the water temperature of all tanks reached the designated temperature was designated as the onset of the cold stress experiment. To ensure the accuracy and stability of the water temperature, a thermometer was used to monitor the water temperature three times a day.

The animals in the three groups were sampled at 0 (before the cooling experiment), 1, 2, 4, 8, and 16 days post-cold-stress (dps). At each timepoint, a total of 24 turtles were sampled to obtain both plasma and splenic tissues for transcriptomic and metabolomic analysis in each group (n = 24). The animals were anesthetized with 1 g/L tricaine methanesulfonate (MS-222) solution immediately before the sampling. Then, the blood samples were acquired from the neck–chest fracture section of the turtles into sterile tubes rinsed with heparin. After the blood was collected, the turtles were dissected quickly to obtain the spleen tissues, which were quickly snap frozen and stored in liquid nitrogen for analysis of the transcriptome and metabolome.

### 2.3. Plasma Biochemical Parameters

To mitigate the effect of individual differences on the accuracy of results, blood from 8 individuals was pooled into one sample (n = 3 per group). The plasma supernatant was separated, centrifugated at 4000× *g* for 20 min at 4 °C, and then kept at −80 °C for analysis of biochemical parameters.

The activities of plasma catalase (CAT, A007-1-1), superoxide dismutase (SOD, A001-3-2), and glutathione peroxidase (GSH-Px, A005-1-2) were assayed using test kits (Nanjing Jiancheng Bioengineering Institute, Nanjing, China). The procedures were carried out guided by the manufacturer’s protocols.

### 2.4. Transcriptomic Analysis of Spleen

T-SOD, CAT, and GSH-Px activities revealed that individuals in the T7 group at 4 dps had the most obvious physiological variation compared with the CG. In order to maximize the discrepancy caused by cold stress, the spleens in the CG and T7 group at 4 dps were chosen for transcriptomic and metabolomic analyses. The splenic tissues from four turtles were pooled into one sample. Three pooled samples (n = 3) in each of the CG and T7 groups at 4 dps were used for transcriptomic analysis. The spleen RNA was extracted from the mixed samples utilizing the TRIzol reagent (Invitrogen Life Technologies, Waltham, MA, USA). The integrity of RNA was assessed by 1% agarose electrophoresis, while the quality and concentration were detected with a NanoDrop spectrophotometer (Thermo Scientific, Waltham, MA, USA).

The constructed RNA libraries were paired-end sequenced on the NovaSeq 6000 platform (Illumina, San Diego, CA, USA) by Suzhou PANOMIX Biomedical Tech Co., Ltd. (Suzhou, China). After filtering out the adapter and low-quality reads, the clean reads were mapped to the reference genome of *P. sinensis* (https://www.ncbi.nlm.nih.gov/datasets/genome/GCF_000230535.1/ (accessed on 24 July 2012)) and deposited in the Short Read Archive (SRA) of NCBI with BioProject accession number PRJNA1185615. The gene expression levels were calculated as FPKM (fragments per kilobase per million mapped fragments). The differentially expressed genes (DEGs) between the two groups were identified with |log_2_ (fold change)| ≥ 1.0 and adjusted *p*-value < 0.05. All DEGs were mapped to gene ontology (GO) and Kyoto Encyclopedia of Genes and Genomes (KEGG) public databases to find out their biological functions.

### 2.5. Metabolomic Analysis of Spleen

Spleens of the CG and T7 groups at 4 dps with five pooled samples in each group (n = 5) were used for metabolomic analysis. Approximate 50 mg of each sample was homogenized with 1 mL mixed solution containing acetonitrile and methanol (acetonitrile–methanol–water = 2:2:1). The samples in the mixed solution were vortexed for 20 s, homogenized at 50 Hz for 120 s, sonicated for 5 min, and then kept in an ice-water bath. Then, the solution was freeze centrifuged at 12,000 rpm for 10 min to obtain the supernatant. The 60 µL supernatant was analyzed with a liquid chromatography–tandem mass spectrometry (LC-MS) system. The liquid chromatography (LC) was carried out in a Thermo Vanquish system (Thermo Fisher Scientific, Waltham, MA, USA). Mass spectrometry was operated on a Thermo Q Exactive HF-X mass spectrometer (Thermo Fisher Scientific, Waltham, MA, USA) in positive and negative polarity modes. The parameter conditions of LC and MS were set as in the previous method [26].

The raw data of LC-MS were converted to mzXML format using the ProteoWizard software package (ver. 3.0) [27]. The XCMS program was used to analyze peak identification, filtration, and alignment. The metabolomic data were annotated with multiple public databases, namely HMDB (https://www.hmdb.ca/ (accessed on 5 July 2024)), Metlin (https://metlin.scripps.edu/index.php (accessed on 5 July 2024)), Massbank (https://www.massbank.jp/ (accessed on 5 July 2024)), LIPID MAPS (https://lipidmaps.org/ (accessed on 5 July 2024)), mzCLOUD (https://www.mzcloud.org/ (accessed on 5 July 2024)), and the BioNovoGene database (http://www.bionovogene.com (accessed on 5 July 2024)). Furthermore, the multivariate statistical analysis (MSA) of metabolites was achieved with partial least squares discriminant analysis (PLS-DA) and orthogonal partial least squares discriminant analysis (OPLS-DA) using the R language package. In order to screen the differentially expressed metabolites (DEMs) between the CG and T7 groups, the threshold parameters of MSA were set as projections of important variables (VIPs) > 1 and *p* < 0.05. The DEMs were mapped to the KEGG database to find the functional pathways.

### 2.6. Combined Analysis of the Transcriptome and Metabolome

The Pearson model was established to evaluate the correlation between the DEGs and DEMs with the Pearson correlation coefficient (PCC) and the relevant *p* value. Parameters of |PCC| > 0.80 and *p* < 0.05 were considered to be significantly correlated. Then, the DEGs and DEMs were mapped to the KEGG database to reveal the significantly regulated pathways. Finally, the network of the DEGs and DEMs was described to reveal the potential immune pathways of the spleen regulated by low temperature.

### 2.7. Validation of Transcriptomic Data

To verify the reliability of the transcriptomic data, a total of 17 DEGs were selected for quantitative real-time PCR (qRT-PCR). The primers for the qRT-PCR are shown in Table S1. The cDNA used in the qRT-PCR validation was the same batch of cDNA synthesized in the transcriptome. The 20 μL reaction volume of each sample contained 2 μL of cDNA, 10 μL of 2 × SYBR Green Master Mix (Takara, Dalian, China), 4 μM of each primer, 0.4 μL of ROX reference dye, and RNase-free water to a final volume of 20 μL. The reaction system was run in an ABI StepOnePlus System (Applied Biosystems, Foster City, CA, USA) with the following program: 95 °C for 5 min, followed by 40 cycles of 95 °C for 5 s and 60 °C for 30 s. Each sample was run with three technique replicates. The β-actin gene was chosen as the reference gene. The 2^−△△CT^ method [28] was used to calculate the fold changes in mRNA levels in the T7 group relative to those in the CG.

### 2.8. Statistical Analysis

The plasma biochemical data were shown as mean ± standard error (SE). The significant differences in these results among the three groups at the same timepoint were evaluated by one-way analysis of variance (ANOVA) followed by Duncan’s post hoc test. *p* < 0.05 was considered to be statistically significant. The correlation between transcriptomic and qRT-PCR data was evaluated by Pearson R^2^ values and plotted with the GraphPad Prism 9.0 software (GraphPad Software Inc., La Jolla, CA, USA). The IBM SPSS 21.0 software (Armonk, New York, NY, USA) was utilized for statistical analysis.

## 3. Results

### 3.1. Changes in Plasma Antioxidant Enzymes Under Cold Stress

**Figure 1:**
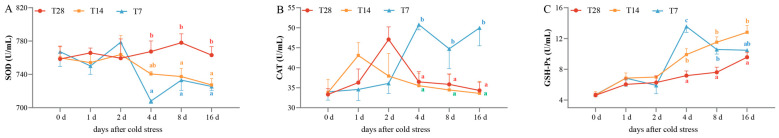
Effects of cold stress on the activities of three plasma antioxidant parameters. (**A**) Superoxide dismutase (SOD) activity. (**B**) Catalase (CAT) activity. (**C**) Glutathione peroxidase (GSH-Px) activity. All data are represented as mean ± SE (n = 3). Different superscript letters mean significant differences in different groups at the same timepoint (*p* < 0.05). “CG” indicates the control group. “T14” indicates the 14 °C cold stress group. “T7” indicates the 7 °C cold stress group.

The activities of three plasma antioxidant enzymes, including T-SOD, CAT, and GSH-Px, were assayed to evaluate the antioxidant capacity (Figure 1). No significant difference was found in the T-SOD, CAT, or GSH-Px activities among three groups before 4 dps (*p* > 0.05). The T-SOD (Figure 1A) activities in the T14 and T7 groups showed decreasing trends from 4 to 16 dps and were obviously inhibited in comparison with that in the CG from 4 to 16 dps (*p* < 0.05). There was no significant difference in the CAT or GSH-Px activities between the CG and T14 groups from 0 to 2 dps (*p* > 0.05); however, the CAT activity (Figure 1B) in the T7 group reached its highest point at 4 dps and remained higher than that in the other two groups until 16 dps (*p* < 0.05). The GSH-Px activity (Figure 1C) in the T14 group from 4 to 16 dps and that in the T7 group from 4 to 8 dps were remarkably higher than that in the CG (*p* < 0.05). Among the three groups, the GSH-Px activity in the T7 group was highest at 4 dps. In general, the T-SOD, CAT, and GSH-Px activities in the T7 group at 4 dps exhibited the most obvious differences with the other two groups, indicating that the individuals in the T7 at 4 dps had the most obvious physiological variation. In order to maximize the discrepancy between the cold stress group and control group, the spleens in the CG and the T7 group at 4 dps were chosen for transcriptomic and metabolomic analysis.

### 3.2. Effect of Cold Stress on Spleen Transcriptome

The transcriptomic sequencing of the CG and T7 groups at 4 dps was performed with three biological replicates in each group (n = 3). A total of six RNA-seq libraries were generated in the CG and T7 groups at 4 dps. There were 50,432,270 and 48,422,531 raw reads, which were sequenced separately, in the CG and T7 RNA-Seq libraries, respectively (Table S2). Following removal of adaptors and low-quality reads, 49,468,643 and 47,479,670 clean reads were obtained in the CG and T7 groups, respectively. Genes were matched with the genome of the Chinese soft-shelled turtle, and the average total mapping rates for the CG and T7 groups were 87.63% and 87.16%, respectively. The Q20 and Q30 values in two groups were all above 95%, implying that the sequencing data were qualified for subsequent analysis.

**Figure 2:**
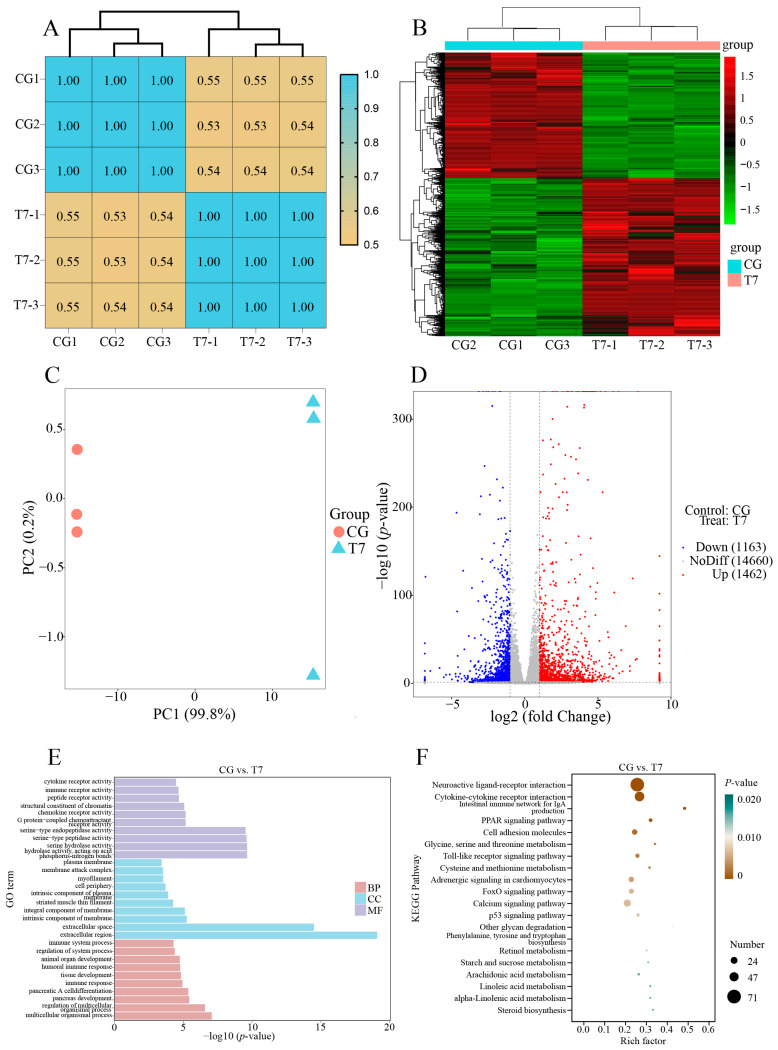
Overall description of the transcriptomic data in the CG vs. T7 comparison. (**A**) The correlation heatmap of all expressed genes in the CG and T7 groups. (**B**) Cluster heatmap analysis of the expressed genes in the CG and T7 groups. (**C**) Principal component analysis (PCA) exhibits the distinction between the gene expression patterns in the CG and T7 groups. (**D**) The volcano plot shows the number of DEGs in the CG vs. T7 comparison. (**E**) GO and (**F**) KEGG enrichment analyses of the DEGs in the CG and T7 comparison. “CG” indicates the control group. “T7” indicates the 7 °C cold stress group. “DEGs” indicates differentially expressed genes.

The correlation matrix of gene expression (Figure 2A) could assess the similarity of the biological triplicates in each group. Our results exhibited that the PCC values of the three biological triplicates per group were 1.00, indicating the high repeatability of the biological triplicates in the same group. The heatmap of hierarchical clustering using significant DEGs (Figure 2B) showed that the CG and T7 groups were separately clustered into two branches, reflecting the distinctive expression profiles between the CG and T7 groups. Principal component analysis (PCA) was conducted to reflect the disparity in transcriptomic results between the CG and T7 groups. In the PCA results (Figure 2C), the first principal component (PC1) was the dominant element in distinguishing the two groups and accounted for 99.8% of the total variation. The volcano plot of DEGs (Figure 2D) revealed that 2625 DEGs in total were identified in the CG vs. T7 comparison, which consisted of 1462 (55.7%) upregulated genes and 1663 (44.3%) downregulated genes.

The functional classification of DEGs was carried out by GO (Figure 2E) and KEGG enrichment analyses (Figure 2F). All DEGs were enriched into three main GO categories, biological process (BP), cellular component (CC), and molecular function (MF) (Figure 2E). The results showed that the top GO terms for DEGs comprised multicellular organismal process (GO:0032501), regulation of multicellular organismal process (GO:0051239), and pancreas development (GO:0031016) for the BP; the extracellular region (GO:0005576), extracellular space (GO:0005615), and intrinsic component of membrane (GO:0031224) for the CC; and hydrolase activity, acting on acid phosphorus–nitrogen bonds (GO:0016825), serine hydrolase activity (GO:0017171), and serine-type peptidase activity (GO:0008236) for the MF. To better understand the biological function of DEGs responding to low-temperature stress, KEGG analysis was carried out for the DEGs in the CG vs. T7 comparison (Figure 2F). There were 152 KEGG pathways enriched by the DEGs, which were divided into 6 categories at level 1 and 28 categories at level 2 of KEGG (Table S3). Among the level 2 KEGG pathways, “carbohydrate metabolism”, “glycan biosynthesis and metabolism”, “lipid metabolism”, and “amino acid metabolism”, occupying 36.1% of the total pathways, were the most enriched categories (Table S3). The top 15 enriched pathways included 9 pathways involved in immune function (Table 1 and Figure 2F), such as “intestinal immune network for IgA production” (*Pigr*, *Il15ra*, *Tnfrsf17*, *Aicda*, *Cd28*, *Itga4*, *Cd40lg*, *Ccr10*, and *Cxcr4*) and “toll-like receptor signaling pathway” (*Mapk10*, *Irf7*, *Mapk13*, *Map2k6*, *Tlr2*, *Tlr5*, *Tlr7*, and *Tlr8*) for the immune system; “cytokine–cytokine receptor interaction” (*Cx3cl1*, *Cx3cr1*, *Cxcl14*, *Cxcr3*, *Cxcr4*, *Cxcr5*, *Ccr4*, *Ccr6*, *Ccr7*, *Ccr10*, *Il12Rb2*, *Il7r*, *Il23r*, *Il15ra*, *Tnfrsf4*, *Tnfsf10*, *Tnfrsf11b*, *Tnfrsf12a*, and *Tnfrsf17*), “neuroactive ligand–receptor interaction” (*Adra1b*, *Adra2A*, *Chrm3*, *Chrm4*, *Chrna3*, *Chrna9*, *Galr1*, *Galr3*, *Glrb*, and *Nmur1*), and “cell adhesion molecules” (*Cd2*, *Cd8b*, *Cd28*, *Cd40Lg*, *Cd226*, *Mag*, *Ocln*, *F11r*, *Cldn2*, *Sdc4*, and *Cdh4*) for signaling molecules and interaction; “calcium signaling pathway” (*Casq1*, *Cacna1b*, *Fgfr3*, *Fgf6*, *Pdgfc*, *Mst1*, *Atp2b2*, *Cacna1I*, *Tnnc1*, and *Ntrk1*) and “FoxO signaling pathway” (*Cdkn1a*, *Mapk10*, *Mapk13*, *Braf*, *Homer2*, *G6pc2*, *Ins*, *Irs1*, *Sgk2*, and *Prkaa2*) for signal transduction; and “p53 signaling pathway” (*Sesn1*, *Sesn2*, *Sfn*, *Gadd45a*, *Serpine1*, *Cdkn1a*, *Cdk1*, *Ccnb1*, *Ccnd2*, and *Pmaip1*) for cell growth and death.

To validate the reliability of the transcriptome, 17 genes were randomly selected to perform an RT-PCR experiment. The PCCs were calculated between the RT-PCR and transcriptomic results (Figure S1). The R^2^ values of the PCCs were 0.99, demonstrating that the mRNA levels from the RT-PCR were consistent with the transcriptomic results, confirming the reliability and accuracy of the transcriptome.

### 3.3. Effect of Cold Stress on Spleen Metabolome

**Figure 3:**
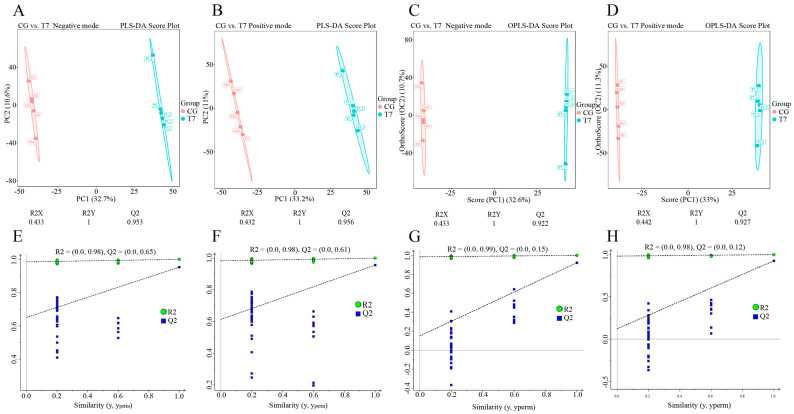
Multivariate statistical analysis of metabolomic data in the CG and T7 groups. Partial least squares discriminant analysis (PLS-DA) score plots with cross-validation of metabolites in negative mode (**A**) and positive mode (**B**). Orthogonal projection to latent structures–discriminant analysis (OPLS-DA) score plots with cross-validation of metabolites in negative mode (**C**) and positive mode (**D**). “R2Y” and “Q2” were two parameters for evaluating the cross-validation model. “R2Y” indicates the explanatory rate, and “Q2” indicates the predictive ability of the PLS-DA and OPLS-DA models. Permutation tests of the PLS-DA models in negative mode (**E**) and positive mode (**F**). Permutation tests of the OPLS-DA models in negative mode (**G**) and positive mode (**H**). “CG” indicates the control group. “T7” indicates the 7 °C cold stress group.

In order to maximize metabolite coverage and detection efficiency, both positive ion mode (POS) and negative ion mode (NEG) were applied in metabolomics analysis (Figure 3). To distinguish the metabolic profiles in the two groups, PLS-DA (Figure 3A,B and Figure 3E,F) and OPLS-DA (Figure 3C,D and Figure 3G,H) models were established for multivariate statistical analysis. The score plots of PLS-DA (Figure 3A,B) and OPLS-DA (Figure 3C,D) showed that the metabolic profiles in both POS and NEG were clearly differentiated between the CG and T7 groups.

To verify the stability and reliability of the PLS-DA and OPLS-DA models, we used R2 and Q2 to evaluate the model performance and perform a permutation test. Cross-validations with the R2Y and Q2 parameters were calculated to assess the stability and reliability of the PLS-DA and OPLS-DA models. The PLS-DA model in positive mode had R2Y = 1 cum and Q2 = 0.956 cum (Figure 3E), and that in negative mode had R2Y = 1 cum and Q2 = 0.953 cum (Figure 3F). Furthermore, the OPLS-DA model in positive mode had R2Y = 1 cum and Q2 = 0.927 cum (Figure 3G), and that in negative mode had R2Y = 1 cum and Q2 = 0.922 cum (Figure 3H). The permutation tests, using the Y-intercept of Q2 as the main parameter, indicated the accuracy of the OPLS-DA model. The Y-intercepts of Q2 were all lower than 0.2, indicating the good accuracy of the OPLS-DA model.

**Figure 4:**
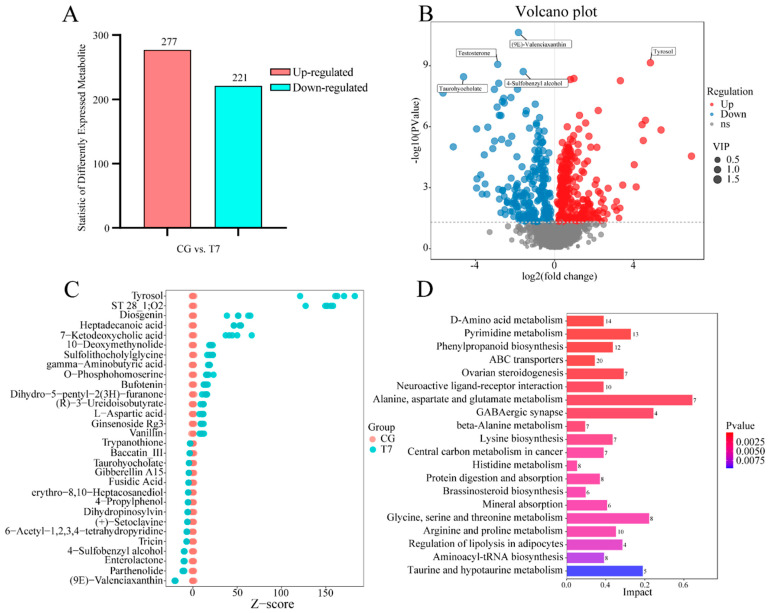
Global analysis of differentially expressed metabolites (DEMs) from the metabolomes of the CG and T7 groups. (**A**) The number of DEMs in the CG vs. T7 comparison. (**B**) Volcano plot of DEMs in the CG vs. T7 comparison. (**C**) Z-score plot showing the top 30 DEMs in the CG vs. T7 comparison. (**D**) KEGG analysis representing the top 20 pathways enriched by DEMs in the CG vs. T7 comparison. “CG” indicates the control group. “T7” indicates the 7 °C cold stress group.

A total of 498 DEMs in both POS and NEG were detected in the CG vs. T7 comparison; these consisted of 277 up-regulated and 221 down-regulated DEMs (Figure 4A,B). Furthermore, a Z-score plot (Figure 4C) showed that esculentic acid, tyrosol, ST 28_1;O2, diosgenin, heptadecanoic acid, 7-ketodeoxycholic acid, erythromycin C, 10-deoxymethynolide, sulfolithocholylglycine, and gamma-aminobutyric acid were the top 10 increased DEMs, while (9E)-Valenciaxanthin, parthenolide, enterolactone, 4-Sulfobenzyl alcohol, tricin, 6-Acetyl-1,2,3,4-tetrahydropyridine, (+)-Setoclavine, dihydropinosylvin, 4-Propylphenol, and erythro-8,10-Heptacosanediol were most obviously declined in T7 compared with CG. The KEGG analysis of the DEMs (Table S3) obtained 218 pathways in both POS and NEG modes. Moreover, 45 pathways were significantly enriched. The 8 amino-acid-metabolism-related pathways were most abundant of the top 20 enriched pathways (Figure 4D), including “D-Amino acid metabolism”, “alanine, aspartate and glutamate metabolism”, “beta-alanine metabolism”, “lysine biosynthesis”, “histidine metabolism”, “glycine, serine and threonine metabolism”, “arginine and proline metabolism”, and “taurine and hypotaurine metabolism”. Multiple pathways related to organism systems were enriched, such as “ovarian steroidogenesis” and “regulation of lipolysis” for the endocrine system, “protein digestion and absorption” and “mineral absorption” for the digestive system, and “GABAergic synapse” for the nervous system. In addition, the DEMs were significantly clustered into “pyrimidine metabolism” for nucleotide metabolism, as well as “phenylpropanoid biosynthesis of biosynthesis” for other secondary metabolites.

**Figure 5:**
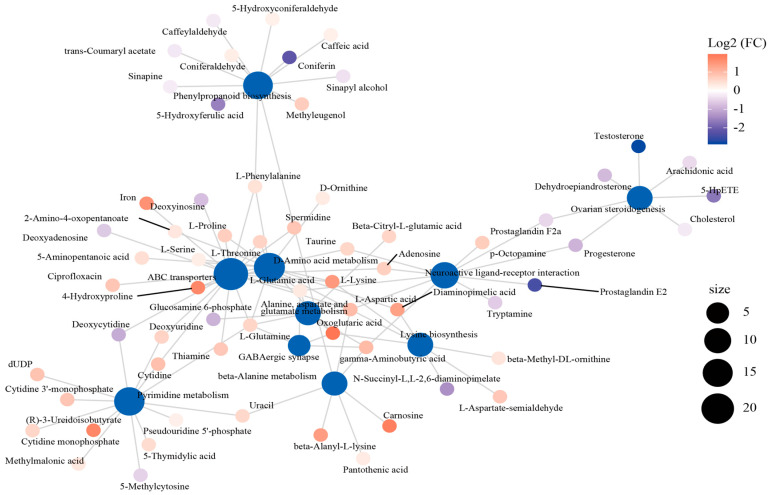
The interactive network connecting the differentially expressed metabolites (DEMs) among the dominant KEGG pathways.

The interaction network was established to describe the relationship between the top 10 enriched metabolic pathways and DEMs (Figure 5). Of these pathways, the “ABC transporter”, “D-Amino acid metabolism”, and “neuroactive ligand–receptor interaction” pathways were in the core position of the network diagram and connected with the other pathways through the shared metabolites. “ABC transporter” was linked with “pyrimidine metabolism” by deoxycytidine, deoxyuridine, and cytidine, as well as with “phenylpropanoid biosynthesis” by L-phenylalanine. In addition, “neuroactive ligand–receptor interaction” was associated with “ABC transporter”, “D-amino acid metabolism”, “lysine biosynthesis”, and “beta-alanine metabolism” through L-aspartic acid.

### 3.4. Joint Analysis of DEGs and DEMs

**Figure 6:**
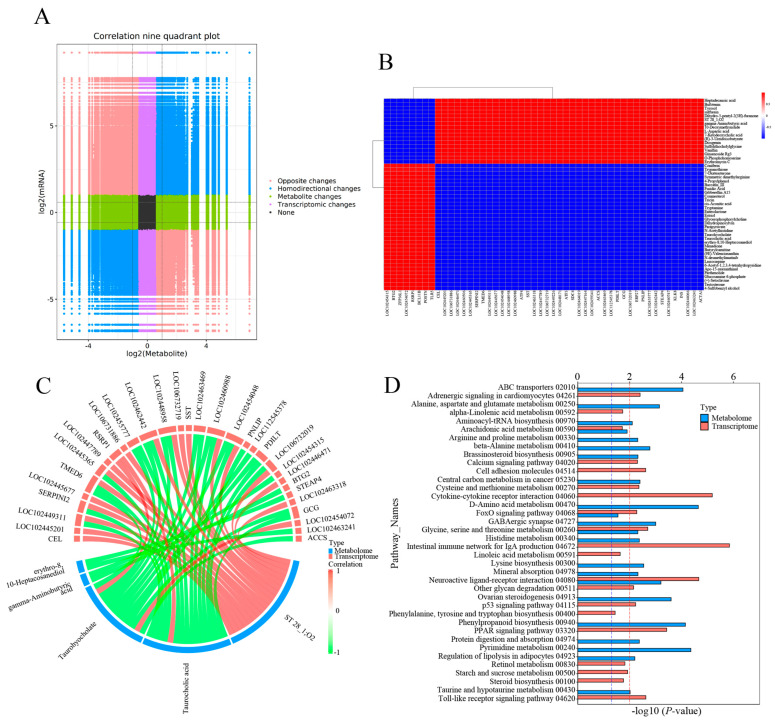
Joint analysis of DEGs and DEMs in the CG vs. T7 comparison. (**A**) Nine-quadrant diagram indicating the correlation between DEGs and DEMs in the CG vs. T7 comparison. (**B**) Heatmap of the correlation between DEGs and DEMs in the CG vs. T7 comparison. (**C**) Chord diagram exhibiting the significant association of DEGs with DEMs in the CG vs. T7 comparison. (**D**) KEGG enrichment analysis of the pathways enriched by DEG and DEMs in the CG vs. T7 comparison. “CG” indicates the control group. “T7” indicates the 7 °C cold stress group. “DEGs” indicates differentially expressed genes. “DEMs” indicates differentially expressed metabolites. The red dashed line indicates the *p* value is 0.01, and the blue dashed line indicates a *p* value is 0.05.

Integrative analysis of the transcriptome and metabolome were performed to establish the association of DEGs and DEMs. A correlation model was constructed to reveal the correlation between DEGs and DEMs (Figure 6). The variation of all DEGs and DEMs in the CG vs. T7 comparison is represented by a nine-quadrant diagram (Figure 6A), with Pearson |r| > 0.80 and *p* < 0.05. The DEMs and DEGs in quadrants 3 and 7 had a positive relationship, while those in quadrants 1 and 9 had a negative relationship. A correlation matrix heatmap (Figure 6B) represents the positive (red) and negative (blue) associations between the top 50 DEMs and DEGs based on PCCs. The heatmap showed that heptadecanoic acid, bufotenin, tyrosol, sulfurein, and L-aspartic acid were negatively correlated with genes such as *Btg2*, *Zfp36l1*, *Rsrp1*, *Bcl11b*, *Postn*, and *Tlr5* but positively associated with genes including *Cel*, *Serpini2*, *Tmed6*, *Atf4*, *Sdc4*, *Accs*, and *Acta1*.

**Figure 7:**
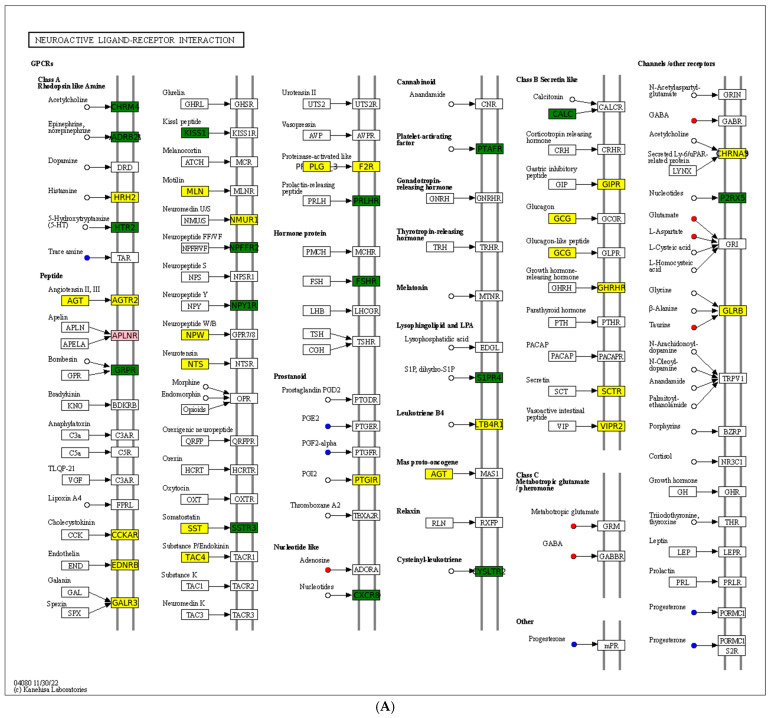

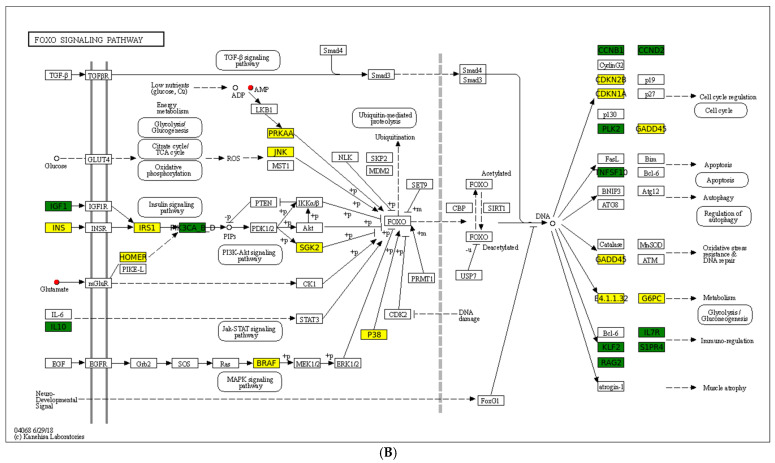
Description of two core pathways responding to cold stress, showing the abundant DEGs and DEMs in the CG vs. T7 comparison. (**A**) Neuroactive ligand–receptor interaction pathway. (**B**) Foxo signaling pathway. The circle represents metabolites and the rectangle represents mRNA. The blue circle indicates downregulated metabolites, the red circle indicates upregulated metabolites, the green rectangle indicates downregulated mRNA, and the yellow rectangle indicates upregulated mRNA.

A chord diagram (Figure 6C) specifically exhibits the association between five DEMs and DEGs. ST 28_1; O2 was positively associated with *Sst*, *Tmed6*, *Serpini2*, and *Cel*. Taurocholic acid was positively associated with *Rsrp1*, while it was negatively related to *Gcg*, *Pdilt*, *Pnlip*, *Tmed6*, and *Serpini2*. Taurohyocholate was positively associated with *Rsrp1*, while it was negatively related to *Accs*, *Gcg*, and *Tmed6*. Next, KEGG analysis of joint DEGs and DEMs was accomplished to find the common pathways, which might be predominant pathways involved in responding to cold stress (Figure 6D). The KEGG histogram showed that four pathways were shared with the DEGs and DEMs, including “glycine, serine and threonine metabolism”, “FoxO signaling pathway”, “arachidonic acid metabolism”, and “neuroactive ligand–receptor interaction” (Figure 7).

## 4. Discussion

The temperature is one of the most critical environmental factors for aquatic ectotherms. Animals exposed to cold stress for a long time can have reduced immune defenses, affecting growth [29]. Considering the severe negative effects of low temperatures, the immune response to acute temperature fluctuations has been studied in multiple species, such as *Litopenaeus vannamei* [30], *Dicentrarchus labrax* [8], and *Trachinotus ovatus* [31]. As the large-scale outdoor farming of the Chinese soft-shelled turtles has rapidly spread in regions of southeast China, the massive death of the turtle has begun to occur during the low-temperature season. It is challenging for turtles to overcome the frequent low temperatures from late autumn to early spring. Therefore, better recognition of the association between cold stress and immunity is essential to find effective strategies and instruct management to improve the survival of this species. In this study, molecular and metabolic biomarkers responding to cold stress in the spleen were identified using transcriptomic and metabolomic analyses. Based on that, the main pathways regulated by low temperature were revealed in the spleen of the Chinese soft-shelled turtle. Investigating how stress modulates immune function may be necessary for disease resistance. In addition, identifying immune mechanisms for coping with cold stress will be significant in advancing the current paradigms of stress signaling.

### 4.1. Cold Stress Affected the Antioxidant Activities

External stimuli can lead to the accumulation of reactive oxygen species (ROS) and thus induce oxidative stress, further destroying biomolecules such as lipids, proteins, and DNA. As typical antioxidant enzymes, T-SOD, CAT, and GSH-Px can clear excessive ROS and protect cells from oxidative stress [32,33]. Our research found that plasma SOD activity was decreased under 14 °C and 7 °C stress after 4 dps in Chinese soft-shelled turtles. Similarly, SOD activity was found to decline under low-temperature stress in *Portunus trituberculatus* [34] and chicken [35]. Moreover, the plasma CAT and GSH-Px activities were increased in turtles exposed to 7 °C cold stress after 4 dps in this study. This result aligned with a previous report on *Macrobrachium rosenbergii*, which showed the CAT and GSH-Px activities were increased under cold stress [36]. Similarly, the GSH-Px activity in a 14 °C group was significantly higher than that in a control group of juvenile hybrid sturgeon [37]. Cold stress has been reported to produce more ROS and induce oxidative stress [38,39]. In our research, the decrease in SOD activity implied that the SOD activity was inhibited, and thus not able to effectively protect the cell from damage due to ROS, under cold stress. Meanwhile, the increased plasma CAT and GSH-Px activities indicated that the CAT and GSH-Px might be the main antioxidant enzymes removing ROS under cold stress for Chinese soft-shelled turtles. Interestingly, the CAT and GSH-Px activities in the control group were not invariable, which might have been attributable to external factors, such as consistent starvation and aggressive behavior between individuals.

Increasing reports have found a dual role of ROS in cell physiology, showing that they not only induce cellular damage but function as important signaling molecules in various biological processes [40]. ROS act as signaling molecules in various cellular functions, such as cell growth, proliferation, differentiation, and apoptosis; immune response; and stress adaptation [41]. For example, ROS can act as signaling molecules to initiate p53 activation in response to DNA damage [42] and regulate the MAPK pathways subject to stressors [43]. Therefore, comprehensive analyses of antioxidant enzymes activities and two omics can reveal the potential role of ROS in regulating the pertinent signaling pathways.

### 4.2. Key Genes and Metabolites Responding to Cold Stress

To resist low-temperature-induced injuries, aquatic organisms regulate the transcription levels of functional genes to adapt to the environmental temperature variation [44]. In this research, abundant DEGs enriched in the “p53 signaling pathway”, “cytokine–cytokine receptor interaction”, and “toll-like receptor signaling pathway” were detected in the 7 °C cold stress group. The p53 signaling pathway is crucial in regulating cell survival and death, primarily through modulating the expression of many target genes involved in cell cycle arrest, DNA repair, senescence, and apoptosis [45]. Our transcriptomic data in the spleen found that *Sesn2*, *Sfn*, *Gadd45a*, *Serpine1*, *Cdkn1a*, and *Pmaip1* of the “p53 signaling pathway” were unregulated after 7 °C cold stress. Furthermore, *Ccnb1*, *Cdk6*, *Igf1*, *Cdk1*, *Rrm2*, *Sesn1*, *Ccnd2*, and *Bcl2* were remarkably decreased. Similar research demonstrated that mRNA expression of some genes in the “p53 signaling pathway” was increased in juvenile hybrid sturgeon after 16 days of cryogenic stress at 14 °C [37]. The genes in the “p53 signaling pathway” were also found to be regulated at the mRNA level in pufferfish after 12 h of low-temperature stress [46]. The DEGs in the “p53 signaling pathway” in this study indicate that cold stress might induce cellular apoptosis in the spleen of the Chinese soft-shelled turtle. These results were consistent with the improved antioxidant activities, implying that cold stress might activate the “p53 signaling pathway” in response to ROS-induced DNA damage.

The “cytokine–cytokine receptor interaction” pathway, as a critical mediator of communication for the immune system, is predominant for host defense against pathogens or stress [47]. In our research, *Epor*, *Tnfrsf12a*, *Il15ra*, *Cxcl14*, *Il31ra*, *Bmp3*, and *Il1rap* were upregulated, while *Cx3cl1*, *Cx3cr1*, *Cxcr3*, *Cxcr4*, *Cxcr5*, *Ccr4*, *Ccr6*, *Ccr7*, *Ccr10*, *Tnfrsf4*, *Tnfsf10*, *Tnfrsf17*, *Tnfrsf11b*, *Il23r*, and *Il7r* in the “cytokine–cytokine receptor interaction” pathway were decreased, after the 7 °C cold stress. A similar report found that the *Cxcr3* and *Cxcl10* in the “cytokine–cytokine receptor” pathway were significantly depressed in the intestines of yellow pond turtles subjected to cold stress at 20 °C for 24 h and 48 h [48]. The abundant DEGs in the “cytokine–cytokine receptor interaction” pathway hinted that this pathway was complicatedly regulated in the spleen under cold stress. Therefore, low temperature might impair immunity in the Chinese soft-shelled turtles via depressing the “cytokine-cytokine receptor interaction” pathway. The “toll-like receptor signaling” pathway, capable of recognizing pathogens and triggering inflammatory and antiviral responses, is considered to have an important part in innate immunity [49]. In the present study, the core genes, including *Map2k6*, *Tlr5*, *Tlr7*, *Tlr2*, and *Tlr8*, were remarkably downregulated in the spleen under 7 °C cold stress. Likewise, *Tlr2* and *Tlr5* were suppressed after low temperature at 10 °C in tilapia [50]. In addition, *Tlr1*, *Tlr2*, *Tlr3*, and *Tlr8* mRNA expression were obviously downregulated after 10 °C cold stress [37]. The suppression of the “toll-like receptor signaling” pathway indicates that the innate immune response to pathogen invasion might be inhibited in the Chinese soft-shelled under cold stress. In general, the downregulation of abundant immune-related genes at mRNA levels after cold stress implied the weakened immune capacity of the turtles. Therefore, it is important to protect turtles from pathogenic infection and take measures to enhance immunity, such as feeding immunostimulants, before the cold stress occurs.

Previous studies have shown that stress and bacterial infection induce profound metabolic reprogramming in aquatic species [51,52]. In this study, 498 DEMs were detected in T7 compared with the control group. Esculentic acid, tyrosol, ST 28_1; O_2_, diosgenin, and heptadecanoic acid were the top upregulated DEMs in the spleen after 7 °C cold stress. Esculentic acid, as a pentacyclic triterpenoid compound, has been reported to have anti-inflammatory activities in mice [53]. Esculentic acid can protect mice against LPS-induced endotoxic shock by regulating inflammatory cytokines, mediators, and COX-2 protein expression [54]. Tyrosol is a phenolic antioxidant that removes ONOO^−^ [55] and O_2_^−^ [56]. Moreover, it also exerts anti-inflammation [57] and neuroprotection functions [58]. Research in rats showed that tyrosol inhibited LPS-induced inflammation by suppressing the NF-κB and p38/ERK MAPK pathways [57,59]. Diosgenin is a steroidal sapogenin that exerts anticancer, cardiovascular protection, antidiabetes, and immunomodulation functions [60,61]. Recent studies have found that diosgenin can alleviate collagen-induced arthritis (CIA) in mice by exhibiting immunosuppressive effects and reducing inflammation [62]. In our research, baccatin III, taurohyocholate, parthenolide, enterolactone, 4-Sulfobenzyl alcohol, tricin, and 4-propylphenol were downregulated in the spleen under cold stress. Baccatin III, a precursor for the semisynthesis of taxol, is widely considered an inactive taxol derivative. It also exerts immunomodulatory activities on MHC-restricted antigen presentation [63]. Taurohyocholate is a kind of bile acid that can be a major component in the bile of lower vertebrates. A study on the invasive turtle found that the taurohyocholate levels in the liver were reduced to cope with low-temperature stress [64]. The alterations of these metabolites in our study might imply that synthesizing anti-inflammatory or immune-related metabolites in the spleen is reprogrammed to cope with the cold-stress-induced inflammation in Chinese soft-shelled turtles.

### 4.3. Key Pathways Responding to Cold Stress

Integrative analysis of DEGs and DEMs was conducted to find the pathways responding to cold stress in the spleen of the Chinese soft-shelled turtle. Four pathways were found to be regulated under low temperature, “glycine, serine and threonine metabolism”, “arachidonic acid metabolism”, “FoxO signaling pathway”, and “neuroactive ligand–receptor interaction”. As the core components of the “glycine, serine and threonine metabolism” pathway, serine and glycine are biosynthetically linked and together supply the essential precursors for the synthesis of proteins, nucleic acids, and lipids that are important for cell growth [65]. In this research, the gene expression (including *Sat1*, *Sardh*, *Cth*, *Gcat*, *Gamt*, *Gldc*, and *Dao*) and metabolite contents (such as L-Aspartic acid, L-Serine, L-Threonine, Creatine, and L-Aspartate-semialdehyde) were significantly changed in the “glycine, serine and threonine metabolism” pathway. Serine/glycine metabolism has been reported to be crucial in sustaining cancer cell survival and rapid proliferation. Excessive activation of serine/glycine biosynthesis triggers tumorigenesis [66]. Furthermore, this pathway is involved in serum-resistant *Escherichia coli*, which can effectively potentiate the serum to kill clinically relevant bacterial pathogens [67]. In brief, our results indicated that cold stress might affect the immunity of the spleen by regulating the “glycine, serine and threonine metabolism” pathway for the Chinese soft-shelled turtle. Considering the crucial function of glycine in this pathway, supplementing ample glycine and serine in diets might be helpful for turtles to resist cold stress.

FOXOs, as members of the FOX family of transcription factors, are conserved evolutionary in most species, with multiple specialized functions in different tissues [68]. In mammals, FOXO-mediated pathways could affect growth factors, oxidative stress, nutrient deprivation, stress resistance, and tumor suppression [69]. Recently, it has been reported the FOXO family can regulate immune function by modulating myeloid cells, including macrophages, DCs, mast cells, monocytes, and granulocytes, which play profound roles in antigen capture, tissue repair, and regulation of effector functions [70]. In addition, this pathway can mediate lymphoid compartments, such as CD4^+^ T cells, CD8^+^ T cells, B cells, and NK cells, which are essential cells in acquired immunity [70]. In the current study, genes including *Cdkn1a*, *Mapk10*, *Mapk13*, *Pck1*, *Braf*, *Homer2*, and *G6pc2*, as well as metabolites comprising adenosine monophosphate and L-Glutamic acid, were remarkably affected in the “FoxO signaling pathway” after cold stress. A similar report in tsinling lenok trout showed that the FoxO signaling pathway in the liver was mediated to cope with high temperature [71]. The FoxO signaling pathway was also involved in regulating high-soybean-meal-induced intestinal inflammation [72]. In general, the current results indicated that the “FoxO signaling pathway” was regulated by cold stress, which further influenced the innate and acquired immunity of spleen tissues. It may be possible to improve the cold tolerance of turtles by adding exogenous adenosine monophosphate and L-Glutamic acid in the feed or upregulating the expression of the *Cdkn1a*, *Mapk10*, *Mapk13*, *Pck1*, *Braf*, *Homer2*, and *G6pc2* genes via gene-editing methods in the daily management process.

The “neuroactive ligand–receptor interaction” pathway is directly associated with neurofunction by binding neuroactive ligands to intracellular receptors [73].The current data showed that DEGs (such as *Glrb*, *Nmur1*, *Sctr*, *Galr3*, *Agt*, *Ltb4r*, *Agtr2*, *Mln*, *Npw*, *Plg*, *Sst*, *Htr1a*, *Gcg*, *Nts*, *Galr1*, *Cckar*, *F2r*, *Adra2A*, and *Ghrhr)* and DEMs (such as L-Glutamic acid, adenosine, taurine, gamma-aminobutyric acid, tryptamine, and progesterone) were detected in the “neuroactive ligand–receptor interaction” pathway. Similarly, transcriptomic analysis of the brain of *Takifugu rubripes* found that the “neuroactive ligand–receptor interaction” pathway was modulated after acute hypoxia stress [74]). Furthermore, this pathway has also been found to be regulated in tilapia under *Streptococcus agalactiae* infection [75] and in sea cucumber under wound healing and early intestinal regeneration [76]. This current data implied the potential role of this pathway in resisting cold-stress-induced injuries in the spleen of the Chinese soft-shelled turtles. Activating this pathway by supplementing external ligands such as acetylcholine, histamine, and 5-hydroxytryptamine in the diet might enhance the ability of turtles to resist cold stress.

## 5. Conclusions

The current study found that plasma CAT and GSH-Px activities were increased to cope with the cold-stress-induced ROS in Chinese soft-shelled turtles. Abundant immune-related DEGs (*Tlr2*, *Tlr5*, *Tlr7*, *Tlr8*, *x3cl1*, *Cx3cr1*, *Cxcl14*, *Cxcr3*, and *Cxcr4*) and DEMs (esculentic acid, tyrosol, and diosgenin) were revealed in the spleen under cold stress by the transcriptome and metabolome, respectively. Conjoint analysis of the two omics discovered that “glycine, serine and threonine metabolism”, the “FoxO signaling pathway”, and the “neuroactive ligand–receptor interaction” were the crucial pathways for the spleen to resist cold stress in the Chinese soft-shelled turtle. During aquaculture management, it may be possible to enhance cold resistance through increasing the mRNA expression of related functional genes, such as *Cdkn1a*, *Mapk10*, *Mapk13*, *Pck1*, *Braf*, *Homer2*, and *G6pc2*, by gene-editing methods. Furthermore, supplementing the metabolites involved in the key pathways, such as L-Glutamic acid, glycine, serine, and histamine, in the diet is another possible strategy to cope with cold stress.

## Figures and Tables

**Table 1:** The differentially expressed genes and pathways of the transcriptomes in the CG vs. T7 comparison.

Category	Pathway	Number of DEGs in Pathway	Up_Gene	Down_Gene
Signaling molecules and interaction	Neuroactive ligand–receptor interaction	Up_number: 39 Down_number: 32	*Glrb*, *Nmur1*, *Sctr*, *Galr3*, *LOC102450564*, *Agt*, *Ltb4r*, *LOC102445365*, *Agtr2*, *LOC106732597*, *Mln*, *LOC102463318*, *LOC112547215*, *Npw*, *Plg*, *Sst*, *Htr1a*, *LOC102455738*, *LOC102445677*, *LOC102450075*, *LOC102462299*, *Gcg*, *Nts*, *LOC102462143*, *LOC102446059*, *Galr1*, *Cckar*, *F2r*, *Adra2A*, *LOC102455240*, *LOC102457024*, *LOC102443518*, *LOC102445111*, *Ghrhr*, *LOC102444212*, *LOC102447960*, *LOC102449550*, *LOC102454403*, *LOC102454640*	*Lpar6*, *Chrna9*, *Kiss1*, *LOC102463720*, *Sstr3*, *P2Rx5*, *Hrh4*, *Npy1Rr*, *Fshr*, *Ptafr*, *LOC102450527*, *LOC102456588*, *Adra1b*, *LOC106731387*, *Htr2b*, *LOC102460605*, *Cysltr2*, *LOC112543526*, *F2*, *S1pr4*, *Chrm3*, *LOC102463328*, *Grpr*, *Chrm4*, *Adrb2*, *LOC106731386*, *Hrh1*, *LOC102462691*, *LOC102445535*, *LOC102450296*, *Npffr2*, *Chrna3*
Signaling molecules and interaction	Cytokine–cytokine receptor interaction	Up_number: 20 Down_number: 30	*Epor*, *LOC102463217*, *Tnfrsf12a*, *LOC102462633*, *Il15ra*, *Cxcl14*, *LOC102454836*, *LOC102455635*, *LOC106732727*, *Il31ra*, *Bmp3*, *LOC106731489*, *LOC102453911*, *LOC102463419*, *LOC102448244*, *LOC102443642*, *LOC102463713*, *Il1rap*, *LOC102450014*, *LOC102445218*	*Cx3cl1*, *LOC102444580*, *Tnfsf10*, *LOC102454894*, *Ccr7*, *Cxcr3*, *Ccr10*, *LOC102458236*, *Tnfrsf11b*, *Cd40lg*, *Edar*, *Tnfrsf4*, *Ccr6*, *Ifng*, *LOC102453719*, *Ccr4*, *LOC102461383*, *Bmp7*, *Cx3cr1*, *Cxcr4*, *Il12Rb2*, *Tnfrsf17*, *Il23r*, *LOC102460133*, *Cxcr5*, *Il7r*, *LOC102451419*, *LOC102459422*, *Eda*, *LOC102457828*
Signaling molecules and interaction	Cell adhesion molecules	Up_number: 12 Down_number: 16	*LOC102458879*, *Mag*, *Ocln*, *F11R*, *LOC102455872*, *Cldn2*, *Sdc4*, *LOC102454797*, *Cdh4*, *LOC102452457*, *LOC102455630*, *Mpz*	*Itga4*, *LOC102462153*, *Cd40Lg*, *Cd2*, *LOC102446295*, *Cd8B*, *LOC112546502*, *Vsir*, *Siglec1*, *Ctla4*, *LOC102456519*, *Cd28*, *Cd226*, *Ptprc*, *Nlgn4x*, *LOC102459758*
Signal transduction	Calcium signaling pathway	Up_number: 22 Down_number: 21	*Casq1*, *Cacna1b*, *Fgfr3*, *Fgf6*, *Pdgfc*, *Mst1*, *Atp2b2*, *LOC102447960*, *LOC102449550*, *Cacna1I*, *Tnnc1*, *Ntrk1*, *LOC102451515*, *Met*, *LOC102460301*, *Pde1c*, *F2r*, *Cckar*, *LOC102459783*, *Camk2a*, *Nos2*, *Slc25a4*	*Cysltr2*, *Mylk3*, *Cxcr4*, *LOC112546502*, *LOC112545842*, *Htr2b*, *Adcy7*, *LOC112547828*, *Camk4*, *Adra1b*, *Ntrk2*, *Ptafr*, *Cd38*, *P2rx5*, *Hrh1*, *LOC106731800*, *Adrb2*, *Grpr*, *Chrm3*, *Pln*, *Gna15*
Signal transduction	FoxO signaling pathway	Up_number: 14 Down_number: 12	*Cdkn1a*, *Mapk10*, *Braf*, *Homer2*, *G6pc2*, *Ins*, *LOC102449535*, *Irs1*, *Sgk2*, *LOC102453637*, *Prkaa2*, *Mapk13*, *Gadd45A*, *Pck1*	*LOC102453719*, *Igf1*, *Klf2*, *Tnfsf10*, *LOC102462482*, *Plk2*, *Ccnd2*, *Rag2*, *Ccnb1*, *Rag1*, *S1pr4*, *Il7r*
Immune system	Toll-like receptor signaling pathway	Up_number: 10 Down_number: 12	*Mapk10*, *LOC102463217*, *LOC102445063*, *LOC102463713*, *Irf7*, *Mapk13*, *LOC102453911*, *LOC106731489*, *LOC102443642*, *LOC102448244*	*LOC102456214*, *Map2k6*, *Tlr5*, *LOC102444580*, *LOC102459334*, *LOC102462482*, *Tlr7*, *LOC102454894*, *LOC102456470*, *Tlr2*, *LOC102460455*, *Tlr8*
Immune system	Intestinal immune network for IgA production	Up_number: 2 Down_number: 14	*Pigr*, *Il15ra*	*Tnfrsf17*, *Aicda*, *LOC106731800*, *Cd28*, *LOC102459422*, *LOC102459758*, *Itga4*, *Cd40lg*, *Ccr10*, *LOC102453719*, *LOC112547828*, *LOC112545842*, *Cxcr4*, *LOC112546502*
Amino acid metabolism	Cysteine and methionine metabolism	Up_number: 10 Down_number: 3	*LOC102445649*, *LOC102456958*, *LOC102461938*, *Psat1*, *Mri1*, *Bcat1*, *Tat*, *Cth*, *Cdo1*, *Mat1a*	*LOC102458451*, *Mtr*, *Mat2a*
Amino acid metabolism	Glycine, serine, and threonine metabolism	Up_number: 12 Down_number: 0	*LOC102451402*, *Psat1*, *Sardh*, *LOC102450327*, *Cth*, *LOC102450557*, *Gcat*, *LOC102445649*, *Gamt*, *Gldc*, *Dao*, *LOC102460961*	*-*
Circulatory system	Adrenergic signaling in cardiomyocytes	Up_number: 19 Down_number: 9	*LOC102450688*, *LOC102456632*, *Tnnc1*, *LOC102451515*, *Scn1b*, *Atf4*, *LOC102460301*, *Tnnt2*, *Ppp2r2c*, *Camk2a*, *Mapk13*, *LOC102456979*, *Cacng3*, *Cacnb2*, *Cacnb4*, *Agtr2*, *Agt*, *Myl3*, *Atp2b2*	*Adcy5*, *Adrb2*, *Pln*, *LOC102455980*, *Cacna2d1*, *Adcy7*, *Adra1b*, *Bcl2*, *Scn4b*
Endocrine system	PPAR signaling pathway	Up_number: 9 Down_number: 10	*LOC102447294*, *Slc27a2*, *Ehhadh*, *Pck1*, *Angptl4*, *Acsl5*, *Fabp3*, *Acsl1*, *Acox2*	*Cd36*, *LOC102444673*, *LOC102457003*, *Rxra*, *Fads2*, *Hmgcs1*, *LOC102448178*, *LOC102447791*, *Fabp7*, *Me1*
Cell growth and death	p53 signaling pathway	Up_number: 8 Down_number: 9	*Sesn2*, *LOC102453637*, *Sfn*, *Gadd45a*, *Serpine1*, *Cdkn1a*, *Pmaip1*, *LOC102462633*	*Ccnb1*, *Cdk6*, *Igf1*, *Cdk1*, *Rrm2*, *Sesn1*, *Ccnd2*, *Bcl2*, *LOC102448042*
Metabolism of cofactors and vitamins	Retinol metabolism	Up_number: 4 Down_number: 6	*LOC102450173*, *Dhrs9*, *LOC102451983*, *LOC102460451*	*LOC102453949*, *Dgat1*, *Aldh1a2*, *LOC102448341*, *LOC102461202*, *Aox1*
Carbohydrate metabolism	Starch and sucrose metabolism	Up_number: 8 Down_number: 1	*Gyg2*, *LOC102447192*, *Si*, *Amy2a*, *G6pc2*, *LOC102447842*, *LOC102459941*, *LOC102460423*	*Treh*
Glycan biosynthesis and metabolism	Other glycan degradation	Up_number: 2 Down_number: 4	*Neu3*, *LOC102443738*	*Neu2*, *Hexb*, *Aga*, *Fuca2*

## Data Availability

All data generated and analyzed during this study are included in this published article. All raw RNA sequencing data have been submitted in the NCBI Sequence Read Archive (SRA) with the BioProject ID PRJNA1185615.

## Supplementary Materials

The following supporting information can be downloaded at: https://www.mdpi.com/article/10.3390/antiox14020217/s1, Table S1: The qRT-PCR primer sequences for *Pelodiscus sinensis*; Table S2: Overview of the sequencing quality of transcriptome; Table S3: KEGG_enrichment of DEGs and DEMs; Figure S1: Pearson correlation analysis of mRNA expression levels of random 17 genes between RNA-seq and real-time PCR results in the CG vs. T7 comparison.
