# Supplementary material for: Molecular Response and Metabolic Reprogramming of the Spleen Coping with Cold Stress in the Chinese Soft-Shelled Turtle (Pelodiscus sinensis)
Source: Antioxidants (Basel). 2025 Feb 14;14(2):217. doi: 10.3390/antiox14020217 (PMC11852077; doi:10.3390/antiox14020217)
Supplement: Supplementary file 1 [file antioxidants-14-00217-s001.zip › Figure S1 Pearson correlation analysis of mRNA expression levels.pdf]

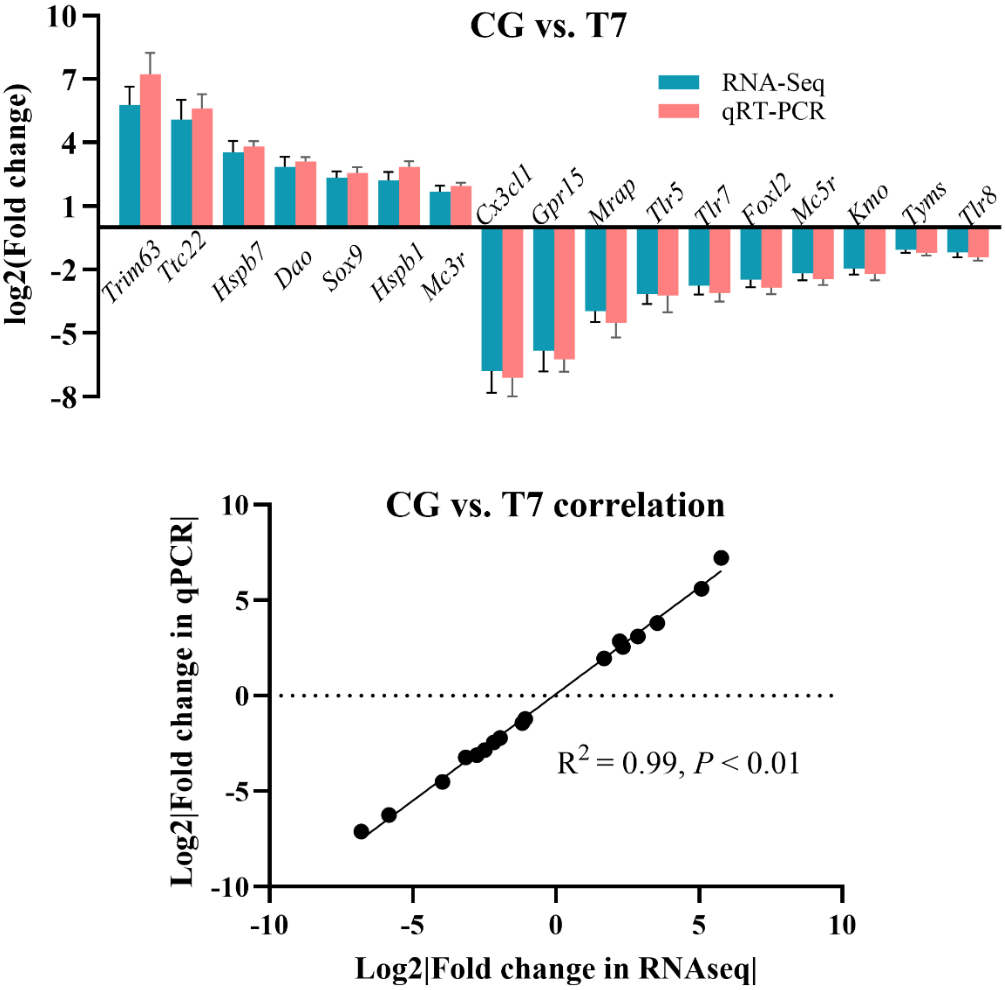

Figure S1. Pearson correlation analysis of mRNA expression levels of random 17 genes between RNA-seq and real-time PCR results in the CG vs. T7 comparison.
