# Supplementary material for: Molecular Response and Metabolic Reprogramming of the Spleen Coping with Cold Stress in the Chinese Soft-Shelled Turtle (Pelodiscus sinensis)
Source: Antioxidants (Basel). 2025 Feb 14;14(2):217. doi: 10.3390/antiox14020217 (PMC11852077; doi:10.3390/antiox14020217)
Supplement: Supplementary file 1 [file antioxidants-14-00217-s001.zip › Table S1 and S2.pdf]

Table S1. The qRT-PCR primer sequences for *Pelodiscus Sinensis*

Gene symbol	Gene name	Forward primer (5'-3')	Reverse primer (5'-3')	NCBI Accession number	product length (bp)
Trim63	E3 ubiquitin-protein ligase TRIM63 isoform X1	ATTACCAGCCCAGCATCATCC	CGCACTTCCGACACAGGTTG	XM_025183240.1	138
Ttc22	tetratricopeptide repeat protein 22	AACACCGGACCAGGACTCATG	CCGCTTCTGTTCGTCATTACC	XM_006133483.3	179
Hspb7	heat shock protein beta-7 isoform X1	AGCCTGTTCGGTGAGGACTTC	ACATCATCGTCACCACCTCCA A	XM_006110466.3	170
Dao	D-amino-acid oxidase	GCACCAGGACCAGGAAGAAG A	ATCACAGCAACACGCATGGC	XM_006126745.3	128
Sox9	transcription factor SOX-9	ACAAAAAGGACCATCCCGACT	TGGCGTTGGGGGAGATGT	XM_025187789.1	114
Hspb1	heat shock protein beta-1	TTCTGCTCCTCCTGGCTCTG	TCTGGGCGTGGCTCTCAAA	XM_025181738.1	195
Mc3r	melanocortin receptor 3	GCCCGCTTGCATGTTAAACG	GAAGGAAGAAAGGCGCCCAA	XM_006129401.3	136
Tyms	thymidylate synthase	ACTCCAGAGGGAACCAC	ACAGCCATCTCCATTTT	XM_014581513.2	135
Tlr8	toll-like receptor 8	CAGCATAGTGTTCTCTTG	ACCGTAGTTCATTCATTAC	XM_014573456.2	277
Kmo	kynurenine 3-monooxygenase isoform X1	TGAACGCTGGATTTGAA	TGGCGAGGTCGGAGATA	XM_006113340.3	132
Mc5r	melanocortin receptor 5	CATGTTCCTCTTGGCTCGTA	GGTCTTCCGCATTTCCTG	XM_006131750.3	295
Foxl2	forkhead box protein L2	AAGCCCCCCTACTCCTATGT	GCCCTTCTTGTTCTTCTCGT	XM_006124992.3	132
Tlr7	toll-like receptor 7	CTTGTCAGGTAATGCTAT	TGTTATCACTCAGGTCTA	XM_014573459.2	161
Tlr5	toll-like receptor 5 isoform X1	TCTCACTGTTCATCTTCA	TATCTATTGCTTGCTTACG	XM_006115600.3	144
Mrap	melanocortin-2 receptor accessory protein	GCTTCCTCCTTCCAGATGTTC	TGTCATCGCCTTTTGGGTT	XM_006126058.3	139
Gpr15	G-protein coupled receptor 15	ATGAGTGGTGACCGCTATCTG G	TTGGTGATGGAGCAGTAGAAG GT	XM_006112270.3	281
Cx3cl1	fractalkine	GGCACAACAGTCTCCCAAGCA	GGACGGTGGCAGATACCTCAG T	XM_025186385.1	256

Table S2. Overview of the sequencing quality of transcriptome

Sample	Raw Reads	Raw Bases	Clean Reads	Clean Data	Clean Reads%	Q20%	Q30%	Clean GC%	TMG%
CG1	53073626	8.01G	52107590	7.83G	98.18	98.55	95.95	45.69	87.64
CG2	47589090	7.18G	46643386	7.01G	98.01	98.38	95.50	45.81	87.38
CG3	50634094	7.64G	49654952	7.44G	98.07	98.42	95.62	45.49	87.87
T7_1	49095174	7.41G	48157932	7.24G	98.09	98.47	95.70	47.48	87.38
T7_2	48454852	7.31G	47489478	7.14G	98.01	98.52	95.87	47.61	87.06
T7_3	47717568	7.20G	46791600	7.04G	98.06	98.46	95.70	47.67	87.03

"CG" indicated the control group. "T7" indicated 7 ℃ cold stress group. "TMG" indicated the ratio of the total clean reads mapped to genome.
